# Book review: Gerlach J, Marusik Y (Eds) (2010) Arachnida and Myriapoda of the Seychelles islands. Siri Scientific Press, 435 pp.

**DOI:** 10.3897/zookeys.182.3105

**Published:** 2012-04-10

**Authors:** Mikhail M. Omelko

**Affiliations:** 1 Far Eastern Federal University, Sukhanova, 8, Vladivostok 690950 Russia; 2 Gornotaezhnaya Station FEB RAS, Gornotaezhnoe Vil., Ussuriyski Dist., Primorski Krai 692533 Russia

The Seychelles islands are located in the Indian Ocean, some 1,500 kilometres east of Africa and northeast of the island of Madagascar. They are particularly interesting from a biogeographical perspective, with ancient affinities to Africa and Asia, recent colonizing species from the Indo-Pacific and modern introductions. Until recently, relatively little was known about the biodiversity of these islands. However, this has changed through the publication of a series of monographs on the Seychelles fauna, presenting the latest information on all the terrestrial and freshwater animals of the islands. In this volume on the Arachnida and Myriapoda of the Seychelles islands, 14 expert scientists from 11 different countries have provided contributions that cover all 433 species of spiders, scorpions, mites, centipedes, millipedes and related groups currently recorded from the islands, including systematic keys, diagnostic illustrations and descriptions for most species, in addition to distribution records.

The volume opens with a brief history of arachnid and myriapod collecting in the Seychelles islands followed by summary data for the numbers of indigenous, endemic and introduced species for all the orders covered and a brief classification scheme for the Arachnida. The following chapters cover the various different orders as follows: Araneae (M.I. Saaristo [deceased], 300 pp., 216 species), Amblypygi (P. Weygoldt, 5 pp.), Opiliones (J. Gerlach, 8 pp.), Scorpiones (L. Prendini, 10 pp.), the smaller arachnid orders including Pseudoscorpiones, Schizomida and Palpigradi (M. Harvey, 10 pp.), Acari: Holothyroidea (J. Gerlach, P. Lehtinen & M. Madl, 7 pp.), Acari: Oribatida (W. Niedbala, 6 pp.), additional Acari (J. Gerlach, 5 pp.), Geophilomorpha (L. Bonato & A. Minelli, 16 pp.), Lithobiomorpha & Scutigerimorpha (P. Stoev & J. Gerlach, 3 pp.), Scolopendromorpha (J.G.E. Lewis, 8 pp.), Diplopoda (S. Golovatch & J. Gerlach, 16 pp.), Symphyla and Pauropoda (J. Gerlach, 6 pp.). Each chapter is fully referenced with its own reference list at the end.

Thus, the majority of the book is devoted to arachnids and spiders in particular, although this is a true reflection of the relative diversity of these groups on the islands. In total, 362 arachnids and 71 myriapod species are documented. In terms of formal taxonomic/nomenclatural changes, one new synonymy is established in the spider family Filistatidae, two new genera of holothyrid mites are described, in addition to three new combinations in the latter group. The book is richly illustrated throughout with clear line drawings, which will permit identification of most of the species covered. The last chapter includes assessments of species conservation status as defined by the International Union for the Conservation of Nature (IUCN) for the indigenous and endemic taxa. There is a comprehensive index.

On the downside, some chapters are more thorough than others, but this is presumably a reflection of the state of knowledge and/or the availability of taxonomic experts with experience in this geographical region. For example, many of the mite species are merely listed as known records without any associated figures, although this still represents a valuable systematic inventory for this group as locality data and citing references are provided. Furthermore, not all Diplopoda are figured and some of the figures provided for this group are rather small. Overall the book is well produced and the editing is tight, although some of the figures and and several of the photographs have not reproduced as well as they may have done, and some e.g. in the Diplopoda chapter are rather small, but they all still serve their intended purpose. Most of the figures are new, although several have been reproduced from other works. It is also the first book to provide complete coverage of the known spider fauna of any single country in the Southern Hemispere. In summary, it provides a comprehensive synthesis of current arachnological and myriapodological knowledge for this previously poorly known region. This review was written by an arachnologist, but I expect the book will be of use to invertebrate taxonomists and biogeographers and deserves a place on the shelves of any serious arachnological or myriapodological taxonomic library.

Price: £60.00 (available directly from the publisher: http://www.siriscientificpress.co.uk)

